# Our First Experience in Using Anatomically Fitted Mesh Implant (Hertra™) During Lichtenstein Repair: A Case Report

**DOI:** 10.7759/cureus.93621

**Published:** 2025-09-30

**Authors:** Mekhaeel Shehata Fakhry Mekhaeel, Andrey Vitalevitch Protasov, Sameh M Salem, Olga Igorevna Mazurova, Abdelrahman Gomaa Zaky Aly Elshliby

**Affiliations:** 1 Operative Surgery and Clinical Anatomy named after I.D. Kirpatovsky, Peoples’ Friendship University of Russia named after Patrice Lumumba (RUDN University), Moscow, RUS; 2 General Surgery, Kirov State Medical University, Kirov, RUS

**Keywords:** inguinal hernia repair, inguinal hernia surgery, lichtenstein technique, mesh fixation, surgical mesh

## Abstract

This article's main goal is to highlight the successful use of a pre-shaped, anatomically fitted mesh implant in Lichtenstein repair. We treated a 65-year-old male patient who experienced a left-sided hernial protrusion for six years, taking into account his concomitant comorbidities: secondary controlled hypertension, chronic superficial gastritis, and lumbar osteochondrosis with an old scar of appendectomy. Lichtenstein repair using a pre-shaped anatomically fitted polypropylene mesh implant with single-suture fixation was the chosen strategy for repair, achieving a concise surgical time along with initial and delayed onset of postoperative complications.

Our objectives during the management of hernia were the use of anatomically fitted mesh implants, seamless fixation, and access to the advantages of anterior hernia repair. Here, we discuss the patient's condition, including medical history, current and local conditions, findings from the investigation, the surgical procedure, and post-surgical developments, additionally elucidating experience-related surgical techniques.

## Introduction

The average cost of a hernioplasty procedure in the United States is approximately $12,500, and that the global incidence of hernias has been steadily increasing over the past 30 years, approaching 33 million cases annually [1]. The global market for hernia repair is expected to reach $6.3 billion in a few years [2]. Given the increasing incidence of hernia and cost of hernia repair, we aimed to improve the results of the gold standard technique of anterior hernia repair by Lichtenstein technique [3].

Polypropylene (PP), the most widely used material for the fabrication of surgical meshes [4], is a non-absorbable, electrostatically neutral, highly hygroscopic, non-polar material with high tensile strength, available as monolayers or multi-layers, coated or uncoated, and in heavy or light forms [5]. The manufacturing technique significantly influences the mesh properties. Traditional knitting creates meshes with mobile joint points, which can lead to high, localized stress concentrations at suture fixation sites, potentially contributing to tissue trauma and post-operative pain. In contrast, advanced techniques like fuse deposition modeling (FDM) 3D printing allow for the creation of pre-formed, anatomically fitted meshes with a more uniform structure [6]. Although this may result in a different tensile profile compared to some knitted meshes, it enables a design that distributes mechanical load more effectively across the implant and tissue interface. This reduces shear forces and pressure on the inguinal nerves, thereby mitigating a major cause of chronic pain. The anatomic design also facilitates quicker and easier implantation, reducing operative time [7].

The use of pre-shaped surgical meshes is preferable to non-pre-shaped fixed sutures due to reduced surgical time, lower post-operative seroma and hematoma formation and urinary retention and, even more importantly, reduced recurrence rates [8,9]. Various hypotheses link the pathogenesis of early post-operative inguinal pain not only to surgical causes but also to tissue trauma from surgical site preparation, mesh inflammatory reactions and mesh fixation, which may explain the less-induced post-operative pain caused by the use of self-adhesive meshes in contrast to the suture.

A significant consideration is cost-effectiveness. While anatomically fitted, pre-formed meshes command a higher initial price than conventional flat polypropylene meshes, analyses show that they provide a superior long-term economic benefit [10,11]. The key drivers of this advantage are: (1) intraoperative efficiency: the pre-formed design eliminates the need for extensive trimming and minimizes the number of sutures required for fixation, significantly reducing operative time and associated costs and (2) reduced complication burden: by promoting better biomechanical integration and reducing nerve entrapment and inflammatory response, these meshes lead to substantially lower the rates of chronic pain, seroma formation, and recurrence. The avoidance of diagnosing and managing these complications results in major cost savings for the healthcare system post-discharge [10,11].

## Case presentation

A 65-year-old male patient was admitted to the outpatient clinic of the clinical hospital No. 85 of the Federal Medico-Biological Agency (FMBA) in Moscow, Russian Federation, complaining of a hernia-bearing disease, gradually increasing in size, in the left groin area for six years. No signs of obstruction or any other hernial complications were reported by the patient. During history taking, the patient clarified that he has a secondary controlled hypertension and his targeted blood pressure level was less than 130/80 mmHg, chronic superficial gastritis, lumbar osteochondrosis, and appendectomy performed by open approach more than 40 years ago. There was no history of allergy.

The patient was alert, comfortable in bed with a level of consciousness on the Glasgow scale of 15/15. The skin had a normal appearance, without pathological rashes and cyanosis. No edema was detected. Visible mucous membranes were pink without pathological rashes. Lymph nodes were neither palpable nor enlarged. An assessment of the musculoskeletal system showed a normal musculature without pathological changes. The patient's height was 170 cm and body weight 80 kg, resulting in a body mass index (BMI) of 27.7 kg/m^2^.

A physical assessment was made. On reviewing the respiratory system, it was found that the shape of the chest was normal and it participates in breathing evenly. Palpation was painless and the percussive sound was pulmonary upon lung auscultation. Respiration was performed evenly throughout the thoracic cavity. Without any wheezing, breathing through the nose was free. Auscultation of the cardiac tones was clear and rhythmic. Abnormal noises were not detected.

The palpation of abdominal organs showed that the liver was along the edge of the costal margins, painless, and not palpable. The gallbladder and spleen were not palpable. Symptoms of peritoneal irritation were not determined. The assessment of the nature of the stools and the frequency of defecation showed that the stool was normal, without pathological inclusions, and passed one to two times a day. No pathological changes were detected in the genitourinary system. Kidneys were not palpable and painless. An assessment of the nature of urination showed that it was free and painless. On assessing vital body signs, body temperature was 36.6°C, heart rate 74 beats/min, blood pressure 125/85 mmHg and respiratory rate 17/min. On local examination, the hernial protrusion was found in the left inguinal region, freely adjustable into the abdominal cavity, painless with normal covering skin. The outer inguinal ring was up to 2.5 cm.

The results of investigations are presented. The complete blood count results are shown in Table 1, the coagulation profile is presented in Table 2, hepato-renal function tests as displayed in Tables 3, 4, and serology for hepatitis B virus (HBV), hepatitis C virus (HCV) and human immunodeficiency virus (HIV) is shown in Table 5.

**Table 1:** Patient’s Complete Blood Count (CBC).

Complete Blood Count (CBC)	Patient’s value	Normal values
Red Blood Cell (RBC) Count	4.9x10^9^/mm^_3_^	4.2 to 6.9x10^9^/mm^3^
Hemoglobin (Hb)	160 g/L	130 to 180 g/L in males 120 to 160 g/L in females
Hematocrit (Hct)	49%	45% to 62% in males 37% to 48% in females
Mean Corpuscular Volume (MCV)	85 micromillimeter	80 to 100 micromillimeter
Mean Corpuscular Hemoglobin (MCH)	30 pg/cell	27 to 32 pg/cell
Mean Corpuscular Hemoglobin Concentration (MCHC)	33%	32% to 36%
White Blood Cell (WBC) Count	6x10^9^/mm^3^	4.3 to 11 x 10^9^/mm^3^
WBC differential		
Neutrophils	7x10^3^/mm^3^	1.8 to 7.8x10^3^/mm^3^
Lymphocytes	3.5x10^3^/mm^3^	0.7 to 4.5x10^3^/mm^3^
Monocytes	0.5x103/mm^3^	0.1 to 1.0 x 10^3^/mm^3^
Eosinophils	0.1 x10^3^/mm^3^	0 to 0.4 x10^3^/mm^3^
Basophils	0	0 to 0.2 x 10^3^/mm^3^
Platelet Count	256x10^9^/mm^3^	150 to 400 x 10^9^/mm^3^
Reticulocytes	0.049x10^9^/mm^3^	1% of total RBC count

**Table 2:** Patient’s coagulation profile

Coagulation Profile	Patient’s value	Normal values
Platelet Count	256 x10^9^/L	150 to 400 x10^9^/L
Prothrombin Time (PT)	15 s	11 to 24 s
International Normalized Ratio (INR)	0.9	0.9 to 1.2
Activated Partial Thromboplastin Time (aPTT)	25 s	22 to 35 s

**Table 3:** Patient’s hepatic function tests PT/INR: Prothrombin time/international normalized ratio.

Hepatic function tests	Patient’s value	Normal values
PT/INR	1	0.9 to 1.2
Serum Albumin	4.0 g/dL	3.5 to 5.0 g/dL
Serum Bilirubin (total)	0.5 mg/dL	0.1 to 1.0 mg/dL
Serum Bilirubin (direct)	0.3 mg/dL	0 to 0.3 mg/dL
Alanine aminotransferase (ALT)	11 U/L	8 to 20 U/L
Aspartate aminotransferase (AST)	8 U/L	8 to 20 U/L
Alkaline phosphatase (ALP)	73 U/L	35 to 100 U/L
Gamma-glutamyl transferase (GGT)	3 U/L	0 to 30 U/L

**Table 4:** Patient’s renal function tests

Renal function tests	Patient’s value	Normal values
Blood urea nitrogen (BUN)	15 mg/dL	7-20 mg/dL
Serum creatinine	0.6 mg/dL	0.6-1.2 mg/dL
Estimated Glomerular Filtration Rate	100 mL/min/1.73 m^2^	>90 mL/min/1.73 m^2^
Albumin to creatinine ratio (ACR)	20 mg/g	<30 mg/g

**Table 5:** Patient’s serology for hepatitis B virus (HBV), hepatitis C virus (HCV) and human immunodeficiency virus (HIV) results HBsAg: Hepatitis B surface antigen

Serology for HBV, HCV	Patient’s value	Normal values
HBsAg	Negative	Negative
Anti-HBc	Negative	Negative
Anti-HBs	Negative	Negative
Serology for HIV	Negative	Negative

Abdomino-pelvic ultrasonographic examination revealed a left inguinal herniation with a hernia sac dimensions of 8x7 cm and width of the outer inguinal ring up to 2.5 cm. The content of hernia was peritoneum. There were no enlarged abdominopelvic organs and no specific abdominal lymphadenopathy. Ascites were not present. ECG was normal. Arterial oxygen saturation was 99% and glomerular filtration rate (GFR) was 53.056. After maintaining controlled blood pressure and obtaining a written informed consent for the operation, the patient was planned for an elective inguinal hernioplasty by Liechtenstein using Hertra™ (Herniamesh, Turin, Italy) mesh implant with spinal anesthesia.

**Figure 1:**
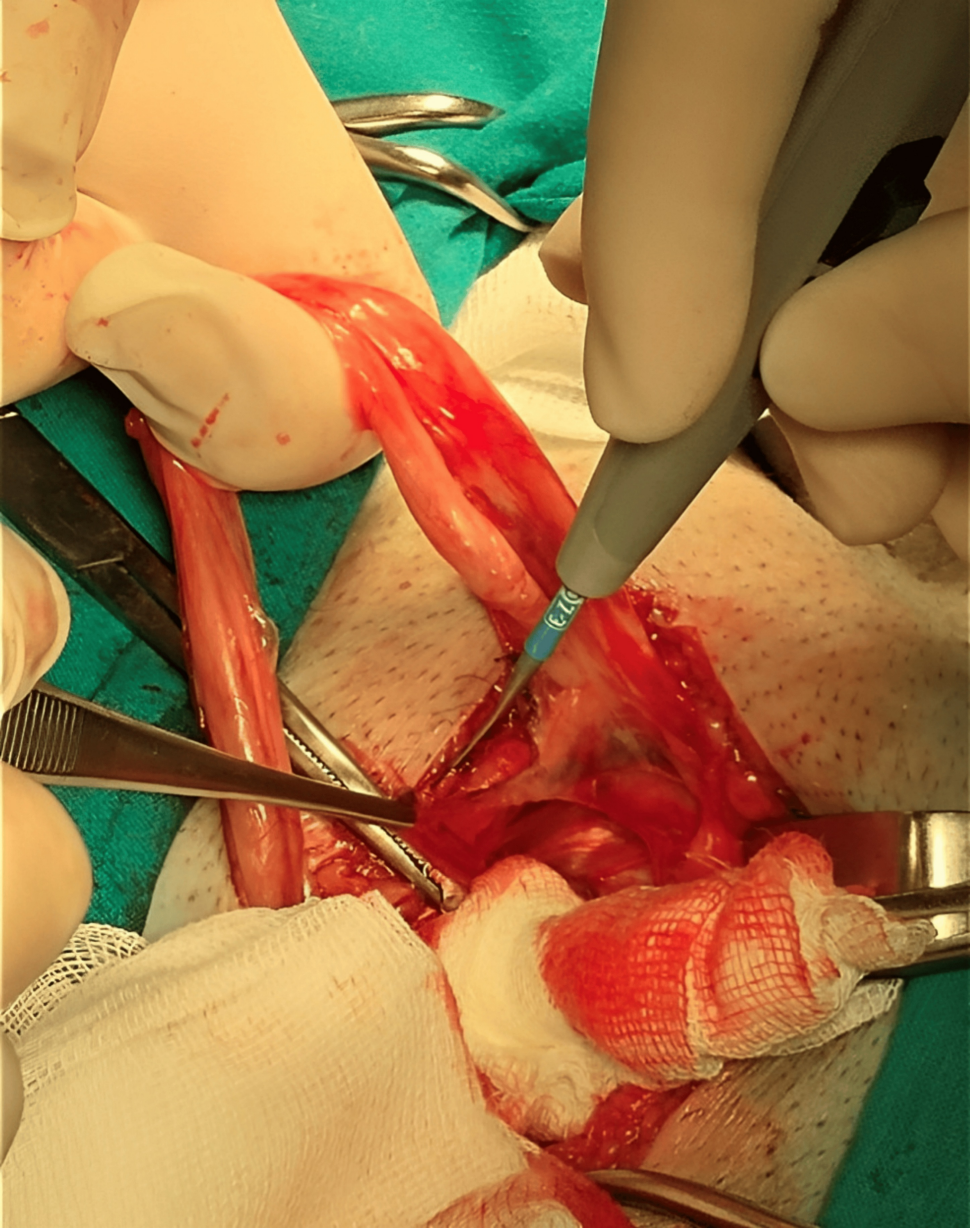
Dissection of the hernial sac.

**Figure 2:**
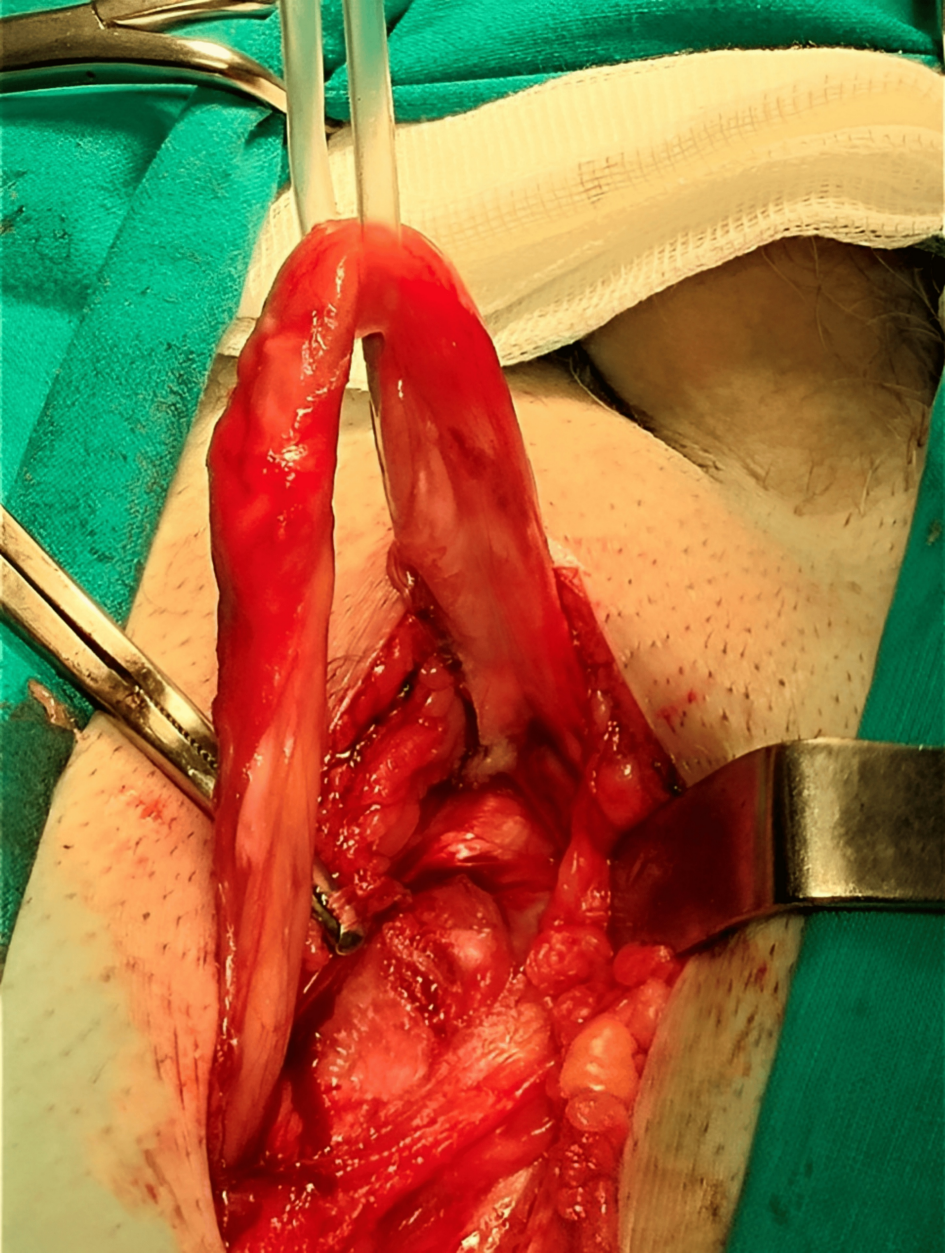
Encircling the spermatic cord using a Penrose.

Cleaning of the inguinal region was performed for two minutes, followed by a 4-cm incision along the inguinal ligament following Langer’s line. For averting postoperative pain, edema, and ecchymosis, abdominal retractors were applied for the prevention of over-retraction. Layer-by-layer dissection was achieved successfully, avoiding injury to the ilioinguinal, iliohypogastric, and genital branch of the genitofemoral nerves through their identification. The ilioinguinal nerve was preserved by careful dissection of the external oblique aponeurosis starting from the external ring along its fibers. Furthermore, local anesthetic was infiltrated around the ilioinguinal nerve and the surgical field lateral to the external oblique aponeurosis to provide pre-emptive analgesia. Herniotomy with high dissection of the hernia sac and complete reduction of the hernial contents back into the abdominal cavity was done as shown in Figure 1. The spermatic cord and the hernia sac were encircled using a Penrose for preserving the genital branch of the genitofemoral nerve as seen in Figure 2.

**Figure 3:**
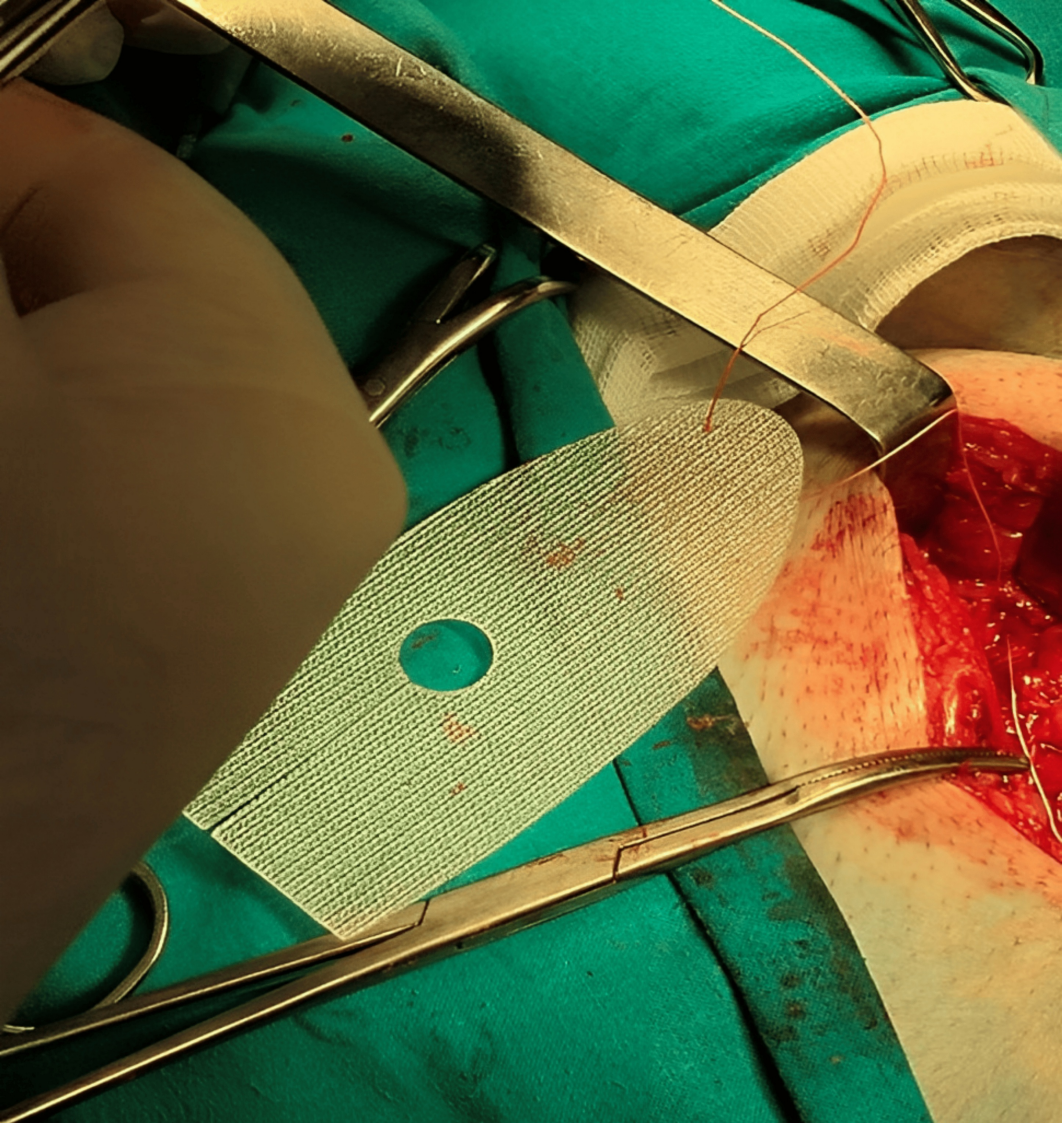
Hertra™ 2 mesh implant.

**Figure 4:**
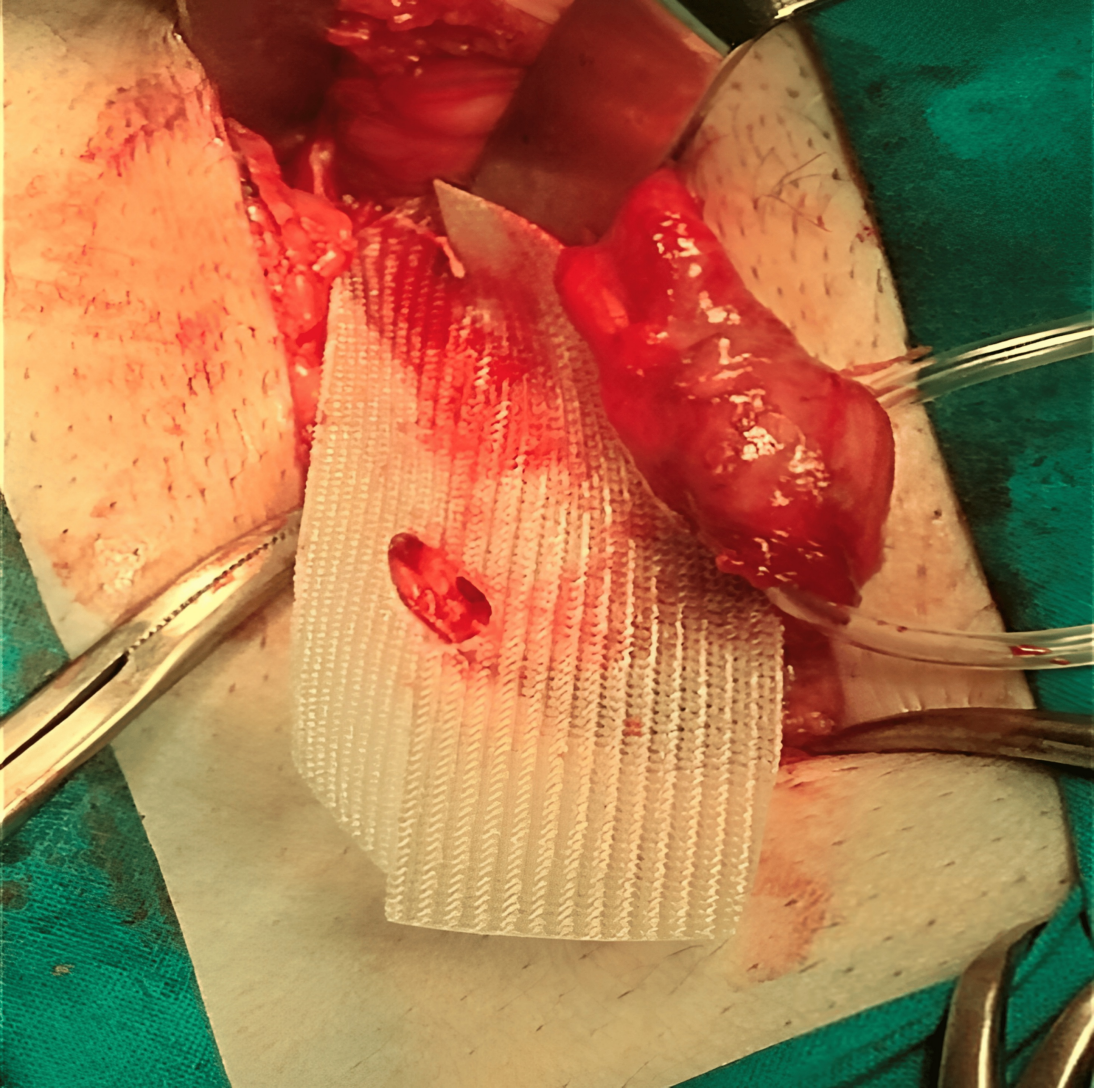
Single-suture fixation to the pubic tubercle.

A semirigid pre-shaped left-sided polypropylene monofilamentous Hertra™ 2 mesh implant, measuring 4.5x10 cm with an eccentric eye and with an anatomically fitted position for the passage of the spermatic cord, was used for repair as seen in Figure 3. No fixation was needed except for a single suture at the pubic tubercle using Dexon zero suture as shown in Figure 4.

**Figure 5:**
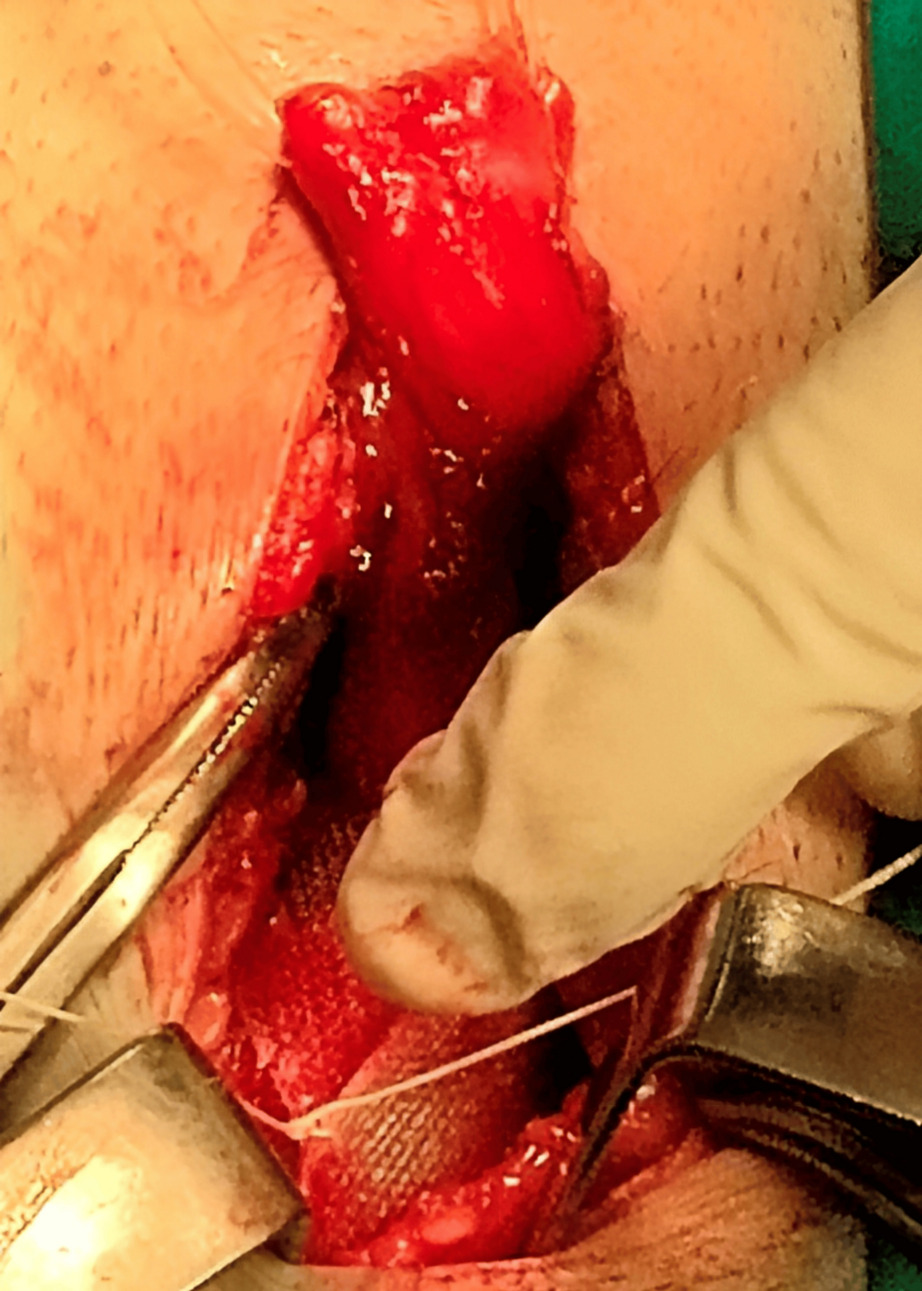
Securing the mesh tails using a single nonabsorbable suture at the level of the internal inguinal nerve.

**Figure 6:**
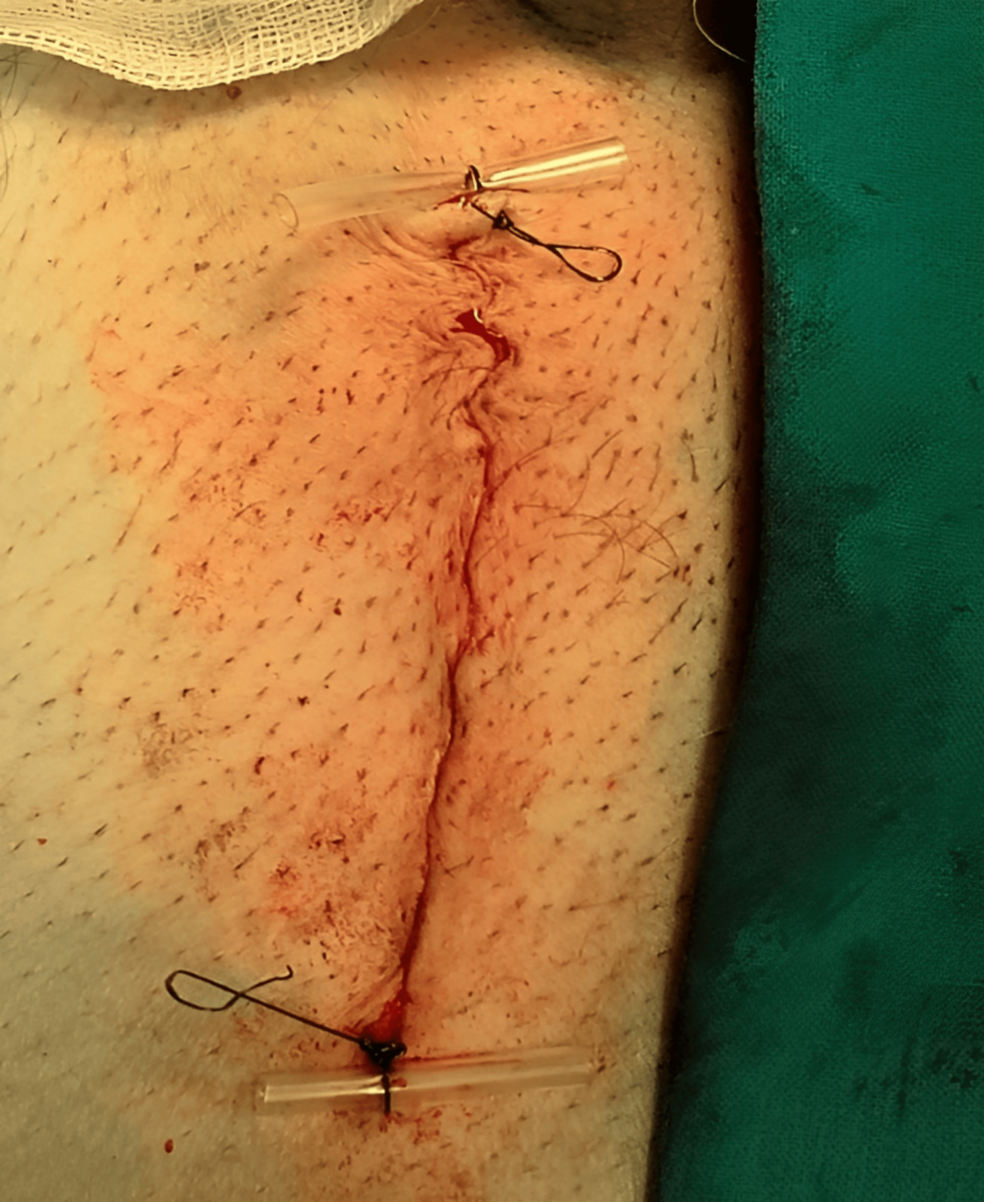
Skin closure with upper and lower markers.

The tails of the pre-splitted mesh encircled the spermatic cord and the internal inguinal ring, followed by securing both ends by a single nonabsorbable suture as seen in Figure 5. The external oblique aponeurosis was reapproximated using absorbable sutures. The wound was closed layer by layer till skin, which was closed by subcuticular suture with upper and lower markers for easier identification of the ends during removal as seen in Figure 6.

The operative time was approximately 30 minutes. During the postoperative period, acute postoperative pain decreased gradually. The surgical dressings were done once daily for a week. During the short-term follow-up, the patient showed no complications, no chronic postoperative pain, no foreign body sensation, and no seroma. There was no recurrence. The follow-up for the first two years was without any complications nor recurrence.

## Discussion

Lichtenstein was performed using semi-rigid polypropylene monofilament mesh Hertra™ 2 (Hertra™​​​​​​​1 and Hertra™​​​​​​​ 2 are pre-adapted for masculine inguinal canal), while Hertra™​​​​​​​ 2A is pre-adapted for feminine inguinal canal geometry with acentric opening with average pore size of 741 microns (min. 389 microns, max. 1083 microns), density of 177 g/m^2^ and sterilized by ethylene oxide [12]. This strategy improved the patient’s surgical output in our experience.

The biologic response to hernia prostheses has a major influence on the operative results and patient’s compliance as well, affecting the compatibility between both the inguinal canal and implanted mesh geometries; flat conventional hernia meshes, fixated with sutures or tacks, induce a poor-quality fibrotic ingrowth, resulting in mesh shrinkage. Furthermore, uncontrolled development of a scar plate can impair movement and may incorporate the ilioinguinal, iliohypogastric, and genital branch of the genitofemoral nerves, resulting in postoperative neuralgia [13]. On the contrary, preformed meshes possess a dynamic responsive behavior as they are designed to adapt the geometry of the inguinal canal; during inguinal movements, preformed meshes induce enhanced biological response with ingrowth of newly formed connective tissue, muscle fibers, and nerves, supporting the contextual development of newly formed arteries and veins, thus inducing the healing process as well [14].

Finally, our patient gained all the above-mentioned benefits, reduced operative time, and absence of complications during both postoperative hospital stays and short-term follow-up periods. Moreover, the patient is following up in the outpatient clinic every six months on a five-year schedule.

## Conclusions

This initial experience with the Hertra™ anatomically contoured, three-dimensional mesh in a Lichtenstein hernioplasty demonstrated notable procedural efficiency and excellent short-term outcomes. The key observation was the mesh's pre-formed design, which facilitated a swift and intuitive implantation process, negating the need for intraoperative trimming and minimizing suture fixation to a single point at the pubic tubercle. This contributed to an operative time of approximately 30 minutes.

In this single case, the patient's postoperative course was unremarkable, with no observed complications such as seroma, chronic pain, or recurrence at the two-year follow-up. These outcomes are consistent with the proposed advantages of such devices, which aim to reduce technical variability, promote physiological force distribution, and minimize nerve irritation.

While these preliminary results are promising, they represent a proof-of-concept from a single instance. The higher initial cost of a pre-formed mesh may be offset by potential gains in operating room efficiency and a reduced complication burden; however, this requires formal health-economic analysis. Therefore, based on this favorable initial experience, we conclude that the Hertra™ mesh is a viable and promising option for open inguinal hernia repair. We strongly recommend and intend to pursue further comparative studies with long-term follow-up to objectively evaluate its advantages over conventional flat meshes in terms of chronic pain, patient satisfaction, and overall cost-effectiveness.
